# Liquiritin from *Radix Glycyrrhizae* Protects Cardiac Mitochondria from Hypoxia/Reoxygenation Damage

**DOI:** 10.1155/2021/1857464

**Published:** 2021-08-06

**Authors:** Vu Thi Thu, Ngo Thi Hai Yen, Nguyen Thi Ha Ly

**Affiliations:** ^1^Center for Life Science Research, Faculty of Biology, VNU University of Science, Vietnam National University, 334 Nguyen Trai, Hanoi, Vietnam; ^2^The Key Laboratory of Enzyme and Protein Technology, VNU University of Science, Vietnam National University, Hanoi, Vietnam; ^3^National Institute of Medical Materials, Hanoi, Vietnam

## Abstract

**Aims:**

The purpose of this study was to evaluate the protective effect of liquiritin (LIQ) from *Radix Glycyrrhizae* on cardiac mitochondria against hypoxia/reoxygenation (HR) injury.

**Methods:**

H9C2 cells were subject to the HR model. LIQ purified from *Radix Glycyrrhizae* (purity > 95%) was administrated to reoxygenation period. Cell viability, mitochondrial mass, mitochondrial membrane potential, reactive oxygen species, and mitochondrial Ca^2^⁺ level were then assessed by using Cell Counting kit-8 and suitable fluorescence probe kits.

**Results:**

LIQ administration remarkably reduced the rate of HR damage via increasing H9C2 cell viability level and preserving mitochondria after HR. Particularly, 60 *μ*M of LIQ posthypoxic treatment markedly reduced cell death in HR-subjected H9C2 cell groups (*p* < 0.05). Interestingly, posthypoxic treatment of LIQ significantly prevented the loss of mitochondrial membrane potential, the decrease in mitochondrial mass, the increase in reactive oxygen species production, and the elevation of mitochondrial Ca^2^⁺ level in HR-treated H9C2 cells.

**Conclusion:**

The present study provides for the first time the cardioprotective of LIQ posthypoxic treatment via reducing H9C2 cell death and protecting cardiac mitochondria against HR damage.

## 1. Introduction

As the “powerhouse” and “apoptosis center” in myocytes, mitochondria have been described to play a key role in the pathogenesis of ischemic heart disease [1, 2]. Mitochondrial dysfunction is disastrous for the heart and has been implicated in hypoxia/reoxygenation injury (HR) [3–6]. During hypoxia, electron transport chain activities are depressed as a result of damage to cardiolipin and an increased H⁺ leak, thereby compromising mitochondrial membrane potential (ΔΨm) and ATP synthesis [1]. Upon reoxygenation, reintroduction of oxygen further compromises mitochondrial malfunction. Phenomena of the malfunctions include an alteration of ΔΨm [7], an excessive generation of reactive oxygen species (ROS), a disruption of Ca^2^⁺ homeostasis [8], an opening of the permeability transition pore [9, 10], and an initiating apoptosis [11]. As mitochondrial abnormality occurring in the reoxygenation period has been identified as a hallmark of ischemic heart disease, targeting mitochondrial protection during reperfusion can improve the recovery of contractile function [4, 12]. On the other hand, preservation of mitochondrial function is essential to limit myocardial damage and subsequently suppresses the progression of ischemic heart disease. In this context, mitochondria are of increasing interest and concern in pharmaceutical and medical research of HR injury [3, 4, 13]. Among the possible mechanisms of cardioprotection, hypoxic postconditioning is one of the most effective mechanisms for protecting cardiomyocytes against HR damage via preventing the mitochondrial structure and function [7, 14], attenuating mitochondrial Ca^2^⁺ accumulation [7] as well as mitochondrial ROS generation [15, 16].

In recent decades, there has been great progress in screening and identifying natural compounds to develop new drugs, which can be used to preserve mitochondrial integrity, limit oxidative stress, and subsequently improve cardiac function in HR [14, 17, 18]. Of those, liquiritin (LIQ) is the main constituent of *Radix Glycyrrhizae* and has been proved to possess various pharmacological activities, such as anti-inflammatory, antioxidant, antiapoptosis, antibacteria [19], and anticancer properties [20–24]. It was previously reported that LIQ exerted protective effects against lipopolysaccharides-induced acute lung injury [25] and high fructose-induced myocardial fibrosis [26]. LIQ was able to resist human corneal endothelial cells apoptosis induced by oxybuprocaine [27] and to interfere with hypaconitine by affecting the CaM expression and Cx43 dephosphorylation in rat cardiac muscle *in vivo* [28]. The combinations of LIQ with other ingredients may also regulate the expression of calcium-regulated proteins to protect rat myocardial cells from damage [29]. Furthermore, extracts from *Glycyrrhiza uralensis* suppressed doxorubicin-induced apoptosis in H9C2 rat cardiac myoblasts [30] and protected the heart against doxorubicin-induced cardiotoxicity [31]. In fact, many traditional medicine formulas containing LIQ have been used to treat cardiovascular diseases, particularly ischemic heart diseases, such as Si-Miao-Yong-An decoction [32], Xuefu Zhuyu decoction [33], YangXinDingJi capsule [18], and ethanol extract of Jaeumganghwa-tang [34]. The studies showed that YangXinDingJi significantly relieved HR damage via increasing the survival of H9C2 cardiomyocytes [33], inhibiting inflammatory cytokine expressions, reducing oxidative stress, and improving contractility in isolated rat cardiomyocytes [18]. Ethanol extract of Jaeumganghwa-tang is relatively more efficacious at protecting against oxidative stress-induced muscle cell death [34]. LIQ single treatment might be a potential agent against cerebral ischemia/reperfusion injury in mice by its antioxidant and antiapoptosis properties [20].

Though LIQ in combination with other constituents or ingredients possessing a potential protective effect on various models was demonstrated, the research evaluating the single treatment of LIQ on the ischemic heart disease model is rare. Up to now, the bioactivity of LIQ in the HR-induced H9C2 model has not been explored yet. Therefore, the present study reports for the first time the cardioprotective effects of LIQ from *Radix Glycyrrhizae* against HR injuries by targeting mitochondria.

## 2. Materials and Methods

The details of each experiment are available in Supplementary Material.

### 2.1. Ethics Statement

All experimental procedures were reviewed and approved by VNU University of Science.

### 2.2. Isolation of Liquiritin (LIQ) from *Radix Glycyrrhizae* (Liquorice Root)

Dried roots of *Radix Glycyrrhizae* (5 kg) were extracted with 96% ethanol (EtOH) under reflux three times. After filtration, the combined EtOH extract was removed under vacuum to obtain a crude extract, which was then suspended in distilled water and partitioned with ethyl acetate (EtOAc) to yield an EtOAc extract (185.0 g). The EtOAc extract was subjected to silica gel column chromatography (CC) and eluted with hexane-EtOAc-methanol (5 : 1 : 0.1, v/v/v), CHCl_3_-acetone-methanol (3 : 1 : 0.1, v/v/v), and CHCl_3_-methanol-water (3 : 1:0.1, v/v/v) to obtain three main fractions (E1–E3). Fraction E3 (24.5 g) was separated on reversed-phase C_18_ (RP-C_18_) CC and eluted with methanol-water (20–70% MeOH, v/v) to afford subfractions (E3A, E3B, E3C). Subfraction E3B was further purified by RP-C_18_ CC with methanol-water (40 : 60 v/v) as an eluted solvent to yield compound LIQ (441.0 mg). The spectral data of LIQ was completely identical with those of LIQ published in the reference [35] (see Supplemental material 1, Table S1-1 and Figures S1.1–S1.7); therefore, the compound LIQ was determined.

### 2.3. Cell Culture

Rat cardiomyocytes, H9C2 cells, obtained from the American Type Culture Collection (ATCC; Rockville, MD, USA), were maintained in Dulbecco's Modified Eagle's Medium (DMEM; Gibco, Invitrogen, Carlsbad, CA, USA) supplemented with 100 *μ*g/ml of penicillin/streptomycin and 10% fetal bovine serum (FBS; Invitrogen) at 37°C with 5% CO₂. The culture medium was changed every 2-3 days. The cells were subjected to HR model, cell viability assay, and mitochondrial relating assays.

### 2.4. Hypoxia/Reoxygenation In Vitro Model and Treatments

H9C2 cells were cultured and subjected to the HR model as described previously [14]. For hypoxic culture, cells were cultured in serum-free low-glucose DMEM at 37°C, 95% N_2_, 5% CO_2_, and 2% O_2_. For reoxygenated culture, cells were cultured under normal conditions following hypoxic culture. After incubation in hypoxic conditions for 6 h, H9C2 cells were then transferred to reoxygenation for 24 h. For the HR-stimulated group, H9C2 cells were reoxygenated without any treatment. For all treatment groups, at the time of reoxygenation, H9C2 cells were separately treated with liquiritin (LIQ, 1.2 ÷ 600 *μ*M) or NecroX-5 (NEC, 10 *μ*M, serves as positive drug control). The stocks of LIQ and NEC were prepared in DMSO and the final concentration of DMSO in the cultured medium was about 0.1%. Cells were cultured under normal conditions without any treatment served as a normal control group.

### 2.5. Cell Viability Assay

Cell viability was assessed by the mitochondrial-dependent reduction of 2-(2-methoxy-4-nitrophenyl)-3-(4-nitrophenyl)-5-(2, 4-disulfophenyl)-2H-tetrazolium, monosodium salt (WST-8) to WST-8 formazan (CCK-8, Dojindo) as described in our last study [14]. For each group, H9C2 cells were seeded in triplicate into 96-well culture plates at a density of 10^4^ cells/well for 24 h. The optimal dose (60 *μ*M) of LIQ was initially determined and chosen for assessing its cardioprotective effects against HR (see Supplementary material 2, Table S2-1, Figures S2-1 and S2-2). By the end of the experimental periods, the cell groups were further incubated for 1–4 h with CCK-8 solution. The absorbance value indicating cell viability was measured at 450 nm using a microplate reader (Microplate Reader, Molecular Devices, USA). The number of living cells in each well was expressed as a value relative to the normal control. Experiments were repeated 6 times.

### 2.6. Measurement of Mitochondrial Mass

Mitochondrial mass was evaluated based on what was previously mentioned [36]. H9C2 cells were seeded at a density of 10^4^ cells/well in 96-well black, glass-bottom plates (CAT. 33196, SPL) or confocal dishes (CAT. 100350, SPL). After HR, NEC, and LIQ60 treatment, H9C2 cells were stained with either MitoTracker Green (0.1 *μ*M, ex/em: 490/516 nm, Invitrogen, USA) or MitoTracker Red (0.1 *μ*M, ex/em: 581/644 nm, Invitrogen, USA) at 37°C for 30 min at room temperature and then washed twice with PBS. The changes in fluorescence intensity reflecting mitochondrial mass are independently assessed by a microplate reader or the ApoTome Fluorescence Microscope (Zeiss, ApoTome). In the assay using the microplate, the mitochondrial mass in each well was expressed as a percentage value relative to the normal control. The cell images were captured using the ApoTome and reconstructed from individual tiles (X:6, Y:9) using ZEN Blue 2.5 software (Carl Zeiss). The MitoTracker intensities (arbitrary unit, AU) of individual cells in captured images were measured. Experiments were performed in triplicate.

### 2.7. Measurement of Mitochondrial Membrane Potential

To measure ΔΨm, H9C2 cells were seeded at a density of 10^4^ cells/well in 96-well black, glass-bottom plates (CAT. 33196, SPL) or confocal dishes (CAT. 100350, SPL). After being subjected to different conditions, cells were stained with 0.1 *μ*M tetramethylrhodamine ethyl ester (TMRE; excitation/emission: 535/570 nm, Invitrogen, USA) for 30 min at room temperature. Next, they were washed twice with PBS before measuring fluorescence intensity using a microplate reader or ApoTome as previously mentioned [14]. The ApoTome-captured images were reconstructed from individual tiles (X:6, Y:9) using ZEN Blue 2.5 software (Carl Zeiss). The TMRE fluorescence intensities (AU) of individual cells in captured images were measured. In the assay using the microplate, the total TMRE intensity in each well was expressed as a percentage value relative to the normal control. Experiments were performed in triplicate.

### 2.8. Measurement of ROS Production

ROS production was evaluated following a previous report [14]. H9C2 cells were seeded at a density of 10^4^ cells/well in 96-well black, glass-bottom plates (CAT. 33196, SPL) or confocal dishes (CAT. 100350, SPL). After being subjected to different conditions, cells were stained with 5 *μ*M 2′,7′-dichlorodihydrofluorescein-diacetate (CM-H₂DCFDA; ex/em 485/525 nm, Invitrogen, USA) at 37°C for 30 min at room temperature to detect changes in mitochondrial ROS levels. After washing twice with PBS 1X, the different fluorescence intensities were measured using the microplate reader. The total H₂DCFDA intensity in each well was expressed as a percentage value relative to the normal control. Experiments were performed in triplicate. In another experimental set, H₂DCFDA-stained cells were captured using the ApoTome and the images were then reconstructed from individual tiles (X:6, Y:9) using ZEN Blue 2.5 software (Carl Zeiss). The fluorescence intensities (AU) of individual cells in captured images were measured. Experiments were performed in triplicate.

### 2.9. Measurement of Mitochondrial Ca^2+^

Mitochondrial Ca^2+^ levels were assed using Rhod-2 AM (5 *μ*M, ex/em: 533/576 nm, Invitrogen, USA) as previously described [7]. H9C2 cells were seeded at a density of 10^4^ cells/well in 96-well black, glass-bottom plates (CAT. 33196, SPL) or confocal dishes (CAT. 100350, SPL) and subjected to the different conditions. After that, the samples were then stained with Rhod-2 AM and then washed twice with PBS 1X. The total intensity value indicating mitochondrial Ca^2+^ was measured by a microplate reader. The different cell groups were also captured using the ApoTome. The images were reconstructed from individual tiles (X:6, Y:9) using ZEN Blue 2.5 software (Carl Zeiss). The Rhod-2 AM intensities of individual cells in captured images were measured. Experiments were performed in triplicate.

### 2.10. Statistical Analysis

Data are presented as means ± standard error of the mean (SEM). Differences between the two groups were evaluated by one-way analysis of variance (ANOVA) and the comparison over time was tested with a two-way ANOVA and Turkey test. Differences with a *p* value ≤0.05 were considered significant.

## 3. Results and Discussions

### 3.1. Liquiritin (LIQ) from *Radix Glycyrrhizae*

**Figure 1:**
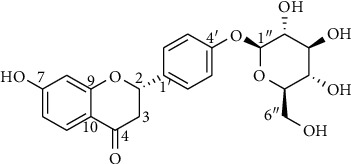
Structure of liquiritin (LIQ) from *Radix Glycyrrhizae*.

Liquiritin (LIQ) was extracted and purified from the root of *Radix Glycyrrhizae* based on the bioassay and chromatographic methods described above. The isolated LIQ compound was identified and had the following characteristics: light yellow amorphous powder, purity >95% (high performance liquid chromatography), and electrospray ionization mass spectrometry: 417.2 [M-H]^−^, 453.1 [M + Cl]^−^ (negative), 419.0 [M + H]^+^ (positive) (Figure 1, Supplemental Material 1). The obtained results showed that *Radix Glycyrrhizae* consists of approximately 0.088 mg/g of the LIQ component, which is consistent with previous reports [35, 37].

### 3.2. LIQ Enhanced H9C2 Cell Viability against HR Injury

**Figure 2:**
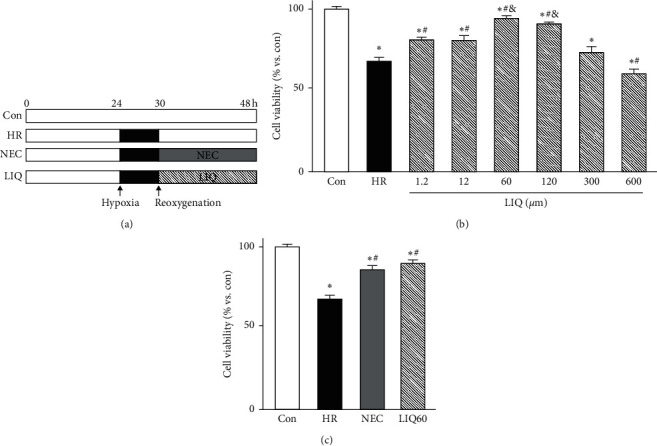
Experimental design and cell viability assay. (a) HR model and LIQ treatment. ((b)-(c)) The graph indicates H9C2 cell viability under different conditions and treatments. Con: normal control; HR: hypoxia/reoxygenation; LIQ: HR + liquiritin (LIQ); NEC: HR + NecroX-5 (10 *μ*M); LIQ60: HR + LIQ at a dose of 60 *μ*M; ^*∗*^*p* < 0.05 vs. Con; ^#^*p* < 0.05 vs. HR; ^&^*p* < 0.05 vs. LIQ at doses of 1.2, 12, 300, and 600 *μ*M, *n* = 6 for each group.

As described above, H9C2 cells were subjected to different conditions and the cell viabilities were measured using the CCK-8 kit (Figure 2). The results showed that the viabilities were dramatically reduced in the HR-exposed cells compared to normal cells (Figures 2(a) and 2(b), *p* < 0.01). In this study, NEC was used as the positive control and showed its efficacy in protecting the cells against HR with a similar value referred to our previous publication [7]. In this study, LIQ at doses of 1.2, 12, 60, 120, and 300 *μ*M strongly prevented the H9C2 cell death from HR. In contrast, LIQ at a dose of 600 *μ*M showed its toxicity to the cardiomyocytes under normal and HR conditions (see Supplemental material 2, Table S2.1, Figures S2.1 and S2.2).

Though the cell viability of other LIQ-treated groups (LIQ at doses of 1.2, 12, 60, 120, and 300 *μ*M) were comparably lower than NEC, supplementation of LIQ during reoxygenation period still significantly minimized the cell death compared to those in HR (66.27 ± 1.53%, *p* < 0.05). Interestingly, among HR-exposed groups, the highest H9C2 viability was presented in the LIQ posthypoxic treatment at a dose of 60 *μ*M (LIQ60). In the LIQ60 group, the cell viability was dramatically increased up to 92.06 ± 2.09% of normal control (100%). It was reported that LIQ increased cell viability via its anti-inflammatory [38], antioxidant, and antiapoptosis activity [20, 23]. The current results further confirmed the safety of LIQ indicated in the previous publication [39]. LIQ has no significant effect on cell viability or hepatotoxicity with different treatments of concentration and duration, suggesting the safety of this compound [39]. However, another study documented that LIQ at the concentrations of 0.1 ÷ 100 *μ*M exerted no cytoprotective effect against cadmium-induced toxicity, whereas liquiritigenin, the hydrolysis product of LIQ or constitutes of *Radix Glycyrrhizae* extract, exerted the cytoprotective effects [40]. Thus, the present data showed that the most effective dose of LIQ against HR damage was about 60 *μ*M. Based on that, we decided to choose LIQ posthypoxic treatment at a dose of 60 *μ*M for further evaluation.

### 3.3. LIQ Preserved Mitochondrial Mass under HR Injury

**Figure 3:**
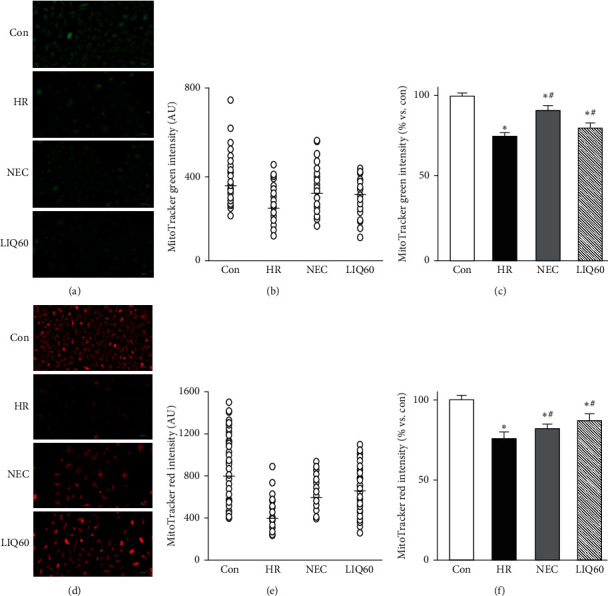
Mitochondrial mass in H9C2 cells subjected to different treatments. (a) Representative images of H9C2 cells stained with MitoTracker Green. (b) The graph indicates MitoTracker green fluorescence intensity per cell (○, open circle) of the representative images. (c) The graph indicates the total MitoTracker green intensity. (d) Representative images of H9C2 cells stained with MitoTracker red. (e) The graph indicates MitoTracker red fluorescence intensity per cell (○, open circle) of the representative images. (f) The graph indicates the total MitoTracker red intensity. AU: arbitrary unit; Con: normal control; HR: hypoxia/reoxygenation; NEC: HR + NecroX-5; LIQ60: HR + LIQ at a dose of 60 *μ*M; ^*∗*^*p* < 0.05 vs. Con; ^#^*p* < 0.05 vs. HR; *n* = 3; scale bar 100 *μ*m.

MitoTracker Green and MitoTracker Red are fluorescent probes, accumulating in mitochondria, and have been used to assess mitochondrial mass or content [36]. To take a closer look at the LIQ roles in mitochondrial mass, we ran the MitoTrackers based experiments and obtained the results as presented in Figure 3. The quantitative results showed either the intensity of individual cells which have been acquired by ApoTome fluorescence microscope (Figures 3(a), 3(b), 3(d), and 3(e)) or the total intensity of wells in the microplate reader (Figures 3(c), 3(f)).

The results showed that the total mitochondrial contents determined by labeling with these two markers were strongly varied under the subjected conditions. We observed that the total MitoTracker Green fluorescence intensity reflecting mitochondrial mass was significantly decreased in HR groups compared to those in the control group (*p* < 0.05). Treatment of either NEC or LIQ60 was able to diminish the HR injury regarding the recovery of mitochondrial mass with the brighter green and red color and with higher intensity values compared to those in HR. The total MitoTracker Green intensity and the total MitoTracker Red values in NEC and LIQ60 groups were significantly higher than those in the nontreated HR group. Additionally, the mitochondrial contents in the subjected groups obtained by quantifying the fluorescent signals per cell were varied in different conditions (Figure 3(a), 3(b), 3(d), and 3(e)). Thus, among HR groups, the lowest mitochondrial contents were presented in HR. The HR groups supplied with either NEC or LIQ60 possessed a higher cell density with the brighter fluorescent color and higher fluorescent values (AU) representing the higher mitochondrial contents. Interestingly, the effects of LIQ on mitochondrial mass, determined in parallel and labelled with the mitochondria-specific MitoTrackers, are almost similar to those in NEC. Here, the obtained results were consistent with the previous study [41], and NEC protected cardiomyocytes via preserving PGC1*α*, the well-known regulator of mitochondrial mass. Therefore, it seems that the preservation effects of LIQ on mitochondrial content constitute a cellular response to compensate for the mitochondria-targeted damage, which was documented in another disease [23]. It was reported that oxidative stress-induced cardiovascular diseases were involved in mitochondrial malfunction and mitochondrial mass. Under the oxidative condition produced by tert-butyl hydroperoxide, the increase in mitochondrial mass in H9C2 cells was linked with the mitochondrial dysfuntion [42]. In addition, total fluorescence intensity values could further confirm the survival cell numbers in each group as demonstrated above (Figure 2). Taken together, a hypoxia-induced decrement in mitochondrial mass associated with a less alive cardiomyocyte numbers could be eliminated by LIQ treatment at the dose of 60 *μ*M.

Given the fact that the ideas of using MitoTracker probes are controversial, in addition to employing these two probes for the purpose of mitochondrial mass, the mitochondrial loading of these dyes shows some sensitivity to Δ*ψ*m [36]. The previous study had also suggested the potential use of MitoTracker to assess the alteration of Δ*ψ*m [43]. Another research had suggested that to interpret appropriate results, technical considerations and parallel complementary assays should be applied [44]. Despite LIQ being effectively targeted on mitochondria mass, the underlying mechanism of mitochondrial function and mitochondrial integrity remained unclear. We assumed that LIQ60 might not only preserve mitochondrial mass but also maintain mitochondrial integrity and function. To test the hypothesis, we carried out the mitochondrial membrane potential assay as described in the next section.

### 3.4. LIQ Posthypoxic Treatment Prevented the Collapse of ∆Ψm from HR Injury

**Figure 4:**
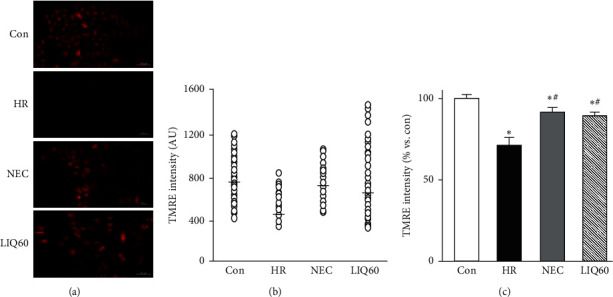
Mitochondrial function in H9C2 cells subjected to different treatments. (a) Representative images of H9C2 cells stained with tetramethylrhodamine ethyl ester (TMRE). (b) The graph indicates fluorescence intensity per cell (○, open circle) of the representative images. (c) The graph indicates the total TMRE intensity; AU: arbitrary unit; Con: normal control; HR: hypoxia/reoxygenation; NEC: HR + NecroX-5; LIQ60: HR + LIQ at a dose of 60 *μ*M; ^*∗*^*p* < 0.01 vs. Con; ^#^*p* < 0.05 vs. HR; *n* = 3; scale bar 100 *μ*m.

Mitochondrial membrane potential is an important index to assess mitochondrial function. A collapse of ∆Ψm reflects a mitochondrial malfunction and consequently leads to cell death [45]. To further assess the role of LIQ60 on mitochondrial function, TMRE was used, according to the manufacturer's instructions. The mitochondrial membrane potential of the experimental groups is presented in Figure 4.

Our data showed that HR significantly induced mitochondrial dysfunction compared to control (*p* < 0.01). Similar to NEC, supplementation of LIQ60 markedly prevented the collapse of ΔΨm against HR injury (*p* < 0.01). There was an insignificant difference in ∆Ψm value, described by TMRE intensities, between the NEC group and LIQ60 group (Figure 4(c), *p* > 0.05). In all groups, the total TMRE intensities were quite matched with the TMRE intensity mean values of individual cells in the presentative images (Figure 4(c)). In consistence with our previous report [7], NEC strongly enhanced mitochondrial function against HR. The present finding suggested that LIQ at a dose of 60 *μ*M showed its ability to prevent the collapse of ∆Ψm in HR-stimulated H9C2. However, regardless of cardiomyocytes, LIQ was able to accelerate the apoptosis via altering ∆Ψm in rheumatoid arthritis pathological condition [46]. LIQ significantly decreased viability and induced apoptosis in the hepatocellular carcinoma cell line by decreasing mitochondrial membrane potential [47]. Here, LIQ was applied during reoxygenation period, so we assumed that the better preservation of mitochondrial membrane potential is closely associated with this compound's ability in modulating mitochondrial mass and eliminating oxidative stress as well as mitochondrial Ca^2+^ overload.

### 3.5. LIQ Decreased Mitochondrial Oxidative Stress Induced by HR Injury

**Figure 5:**
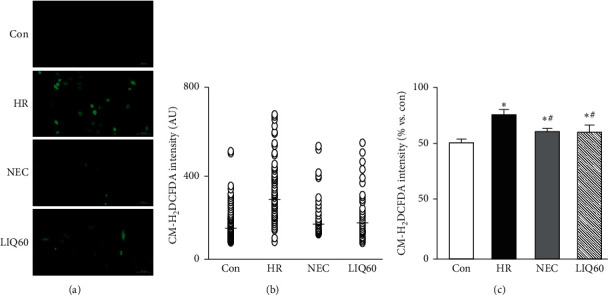
Mitochondrial oxidative stress in H9C2 cells subjected to different treatments. (a) Representative images of H9C2 cells stained with 5-(and-6)-chloromethyl-2′,7′-dichlorodihydrofluorescein diacetate, acetyl ester (CM-H₂DCFDA). (b) The graph indicates fluorescence intensity per cell (○, open circle) of the representative images. (c) The graph indicates the total CM-H_2_DCFDA intensity; AU: arbitrary unit; Con: normal control; HR: hypoxia/reoxygenation; NEC: HR + NecroX-5; LIQ60: HR + LIQ at a dose of 60 *μ*M; ^*∗*^*p* < 0.05 vs. Con; ^#^*p* < 0.05 vs. HR; *n* = 3 ÷ 5; scale bar 100 *μ*m.

The ability of LIQ60 to stabilize the integrity of mitochondrial membrane was further shown in examining ROS production. Here, ROS levels in H9C2 were determined using CM-H_2_DCFDA (Figure 5).

As shown in Figure 5, ROS levels were highly increased under HR conditions compared to those in normal control conditions (*p* < 0.05). Notably, treatment of either NEC or LIQ was able to significantly decrease the HR-induced ROS levels (*p* < 0.01). The CM-H_2_DCFDA intensity mean value of individual cells was almost similar to that counted in total fluorescence intensity. Interestingly, the ability to suppress ROS generation of LIQ seems to be equal to NEC (*p* > 0.05). In fact, NEC is well known as the active compound playing multiple roles and targeting multiple cellular signals. In particular, NEC was previously described as the strong mitochondrial oxidative stress scavenger combating the overproduction of ROS [7, 48]. Thus, the present finding could reconfirm the antioxidative stress of LIQ reported in the previous studies [49, 50]. Earlier studies had also demonstrated the LIQ effectively decreased ROS production in hepatocellular carcinoma cell line [47], skin cells [39], and neuron cells [20]. It was reported that the increase in ROS production was associated with the loss of cardiolipin in lethal cell or the disruption of mitochondrial membrane integrity under ischemia injuries [7, 51]. In this study, the obtained data proved the role of LIQ in protecting H9C2 cells against HR injuries. Thus, the efficacy in maintaining mitochondrial membrane structure and function of LIQ could reinforce for the role of *Radix Glycyrrhizae* extract on protecting cardiomyocytes [17], possibly by targeting the main source of oxidative stress.

### 3.6. LIQ Attenuated Mitochondrial Calcium Level in HR Injury

**Figure 6:**
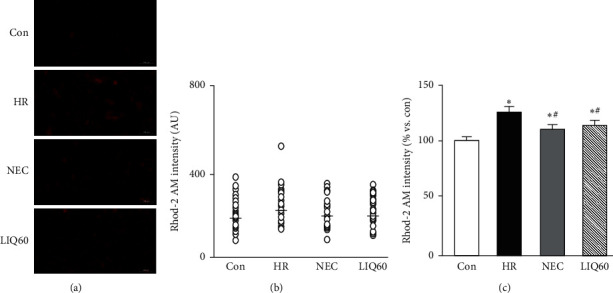
Mitochondrial calcium level in H9C2 cells subjected to different treatments. (a) Representative images of H9C2 cells stained with Rhod-2 AM. (b) The graph indicates fluorescence intensity per cell (○, open circle) of the representative images. (c) The graph indicates the total Rhod-2 AM intensity; AU: arbitrary unit; Con: normal control; HR: hypoxia/reoxygenation; NEC: HR + NecroX-5; LIQ60: HR + LIQ at a dose of 60 *μ*M; ^*∗*^*p* < 0.05 vs. Con; ^#^*p* < 0.05 vs. HR; *n* = 3; scale bar 100 *μ*m.

As a consequence of HR damage, an imbalance in Ca^2^⁺ homeostasis could lead to abnormal contraction of cardiomyocytes, consequently leading to heart malfunction [52, 53]. Of those, mitochondrial Ca^2^⁺ overload could contribute to hypercontraction of cells upon reoxygenation. We found that mitochondrial Ca^2+^ level in HR groups was markedly increased in HR groups compared to the control group. That increase was pronounced in the HR-stimulated H9C2 cells without treatment of either NEC or LIQ60. Supplementation of NEC and LIQ strongly suppressed the elevation of mitochondrial Ca^2+^ level (*p* < 0.05). Interestingly, similar to NEC, HR-induced mitochondrial Ca^2^⁺ increase was strongly attenuated in the LIQ60 group compared with the HR group (Figure 6, *p* < 0.05).

The total Rhod-2 AM intensity (Figure 6(c)) was consistent with the mean Rhod-2 AM intensity value (in AU) of individual myocardiocytes in the representative images of control, HR, NEC, and LIQ60 (Figures 6(a) and 6(b)). Notably, treatment of either NEC or LIQ markedly diminished the HR-induced mitochondrial Ca^2+^ alteration. As previously suggested, the high mitochondrial Ca^2^⁺ level in the HR group finally caused hypercontracture and cardiac cell death; however, NEC treatment dramatically prevented HR-induced Ca^2^⁺ overload, hypercontracture, and cardiac cell death [7]. The observations had suggested that mitochondrial Ca^2⁺^ plays a crucial role in mediating cell death at the first few minutes of reoxygenation as well as during this prolonged period [7, 10]. Of the HR injuries, the reciprocal interactions between mitochondrial Ca^2^⁺ induced ROS overproduction and ROS modulated Ca^2^⁺ overload had been documented. As evidenced above, the cytoprotective effect of LIQ60 could be due to the mitochondrial ROS scavenging activity. Moreover, the effects of LIQ on Ca2⁺ were evidenced in various conditions. In a very recent report, LIQ was presented as a novel inhibitor of transient receptor potential cation channel 1 and transient receptor potential ion channel 1, the Ca^2^⁺ relating channels in lung inflammation injury [25]. LIQ also ameliorated Ca^2^⁺ overload in the model of glutamate-induced cell damage in differentiated pheochromocytoma cells [54]. Additionally, LIQ was able to lower intracellular Ca^2^⁺ in *Phytophthora capsici* [55]. In this study, although the modulation of LIQ60 on mitochondrial calcium uniporter in stabilizing Ca^2^⁺ level was not analyzed yet, the trend and its end effect on this ion could further prove the protective role of LIQ60 on cardiac mitochondria.

## 4. Conclusion

To the best of our knowledge, this is the first study investigating the effect of LIQ on cardiac mitochondria against HR damage. Our results indicated that LIQ effectively increased H9C2 survival, preserved mitochondrial mass, prevented the collapse of ΔΨm, decreased the ROS elevation, and attenuated the overload of mitochondrial Ca^2^⁺. The pilot data might help clarify the myocardial protection role of LIQ against HR injury targeting mitochondria. The new role of LIQ might bring new hopes and potentials for drug development.

## Data Availability

The underlying data supporting the results and the details of each experiment are available in the Supplementary Material.

## Supplementary Materials

Supplementary Materials Supplementation material 1 (S1): Isolation of liquiritin (LIQ) from *Radix Glycyrrhizae* (liquorice root) (RG). Dried roots of liquorice root (5 kg) were extracted with 96% ethanol (EtOH) under reflux three times (each 20 L). After filtration, the combined EtOH extract was removed under vacuum to obtain a crude extract, which was then suspended in distilled water and partitioned with ethyl acetate (EtOAc) to yield an EtOAc extract (185.0 g). The EtOAc extract was subjected to silica gel column chromatography (CC) and eluted with hexane-EtOAc-methanol (5 : 1 : 0.1, v/v/v), CHCl_3_-acetone-methanol (3 : 1 : 0.1, v/v/v), and CHCl_3_-methanol-water (3 : 1 : 0.1, v/v/v) to obtain three main fractions (E1-E3). Fraction E3 (24.5 g) was separated on reversed-phase C_18_ (RP-C_18_) CC and eluted with methanol-water (20–70% MeOH, v/v) to afford three subfractions (E3A, E3B, E3C). Subfraction E3B was further purified by RP-C_18_ CC with methanol-water (40 : 60 v/v) as eluted solvent to yield compound LIQ (441.0 mg). Compound LIQ: yellow powder; mp. 210–212°C; UV (MeOH) *λ*_max_ 217, 275, 311 nm; IR *ν*_max_ (cm^−1^): 3429 (hydroxy), 3329, 2911, 2077, 1607 (carbonyl), 1511, 1446, 1376, 1290, 1233, 1118, 1081, 1044, 997, 889, 834, 632, 531; APCI-MS: *m*/*z* 417.2 [M − H]^−^, 453.1 [M + Cl]^−^ (negative), 419.0 [M + H]^+^ (positive); C_21_H_22_O_9_; ^1^H-NMR (500 MHz, CD_3_OD): *δ*_H_ 7.75 (1H, d, *J* = 9.0 Hz, H-5), 7,46 (2H, d, *J* = 9.0 Hz, H-2′, H-6′), 7.16 (2H, d, *J* = 8.5 Hz, H-3′, H-5′), 6.52 (1H, dd, *J* = 9.0, 2.5 Hz, H-6), 6.39 (1H, d, *J* = 2.5 Hz, H-8), 5.47 (1H, dd, *J* = 13.0, 2.5 Hz, H-2), 4.96 (1H, d, *J* = 7.0 Hz, H-1″), 3.91 (1H, dd, *J* = 12.0, 2.0 Hz, H-6″a), 3.71 (1H, dd, *J* = 12.0, 5.5 Hz, H-6″b), 3.40–3.50 (4H, *m*, H-2″, H-3″, H-4″, H-5″), 3.06 (1H, dd, *J* = 17.0, 13.0 Hz, H-3a), 2.75 (1H, dd, *J* = 17.0, 2.5 Hz, H-3b); ^13^C-NMR (125 MHz, CD_3_OD): *δ*_C_ 193.2 (C-4), 166.8 (C-7), 165.4 (C-9), 159.2 (C-4′), 134.4 (C-1′), 129.9 (C-5), 128.8 (C-2′, C-6′), 117.8 (C-3′, C-5′), 115.0 (C-10), 111.8 (C-6), 103.9 (C-8), 102.2 (C-1″), 80.7 (C-2), 78.2 (C-5″), 78.0 (C-3″), 74.9 (C-2″), 71.4 (C-4″), 62.5 (C-6″), 45.0 (C-3). The spectral data of LIQ was completely identical with those of liquiritin published in the reference [35] (see Table S1.1, Figure S1.1–7); therefore, the compound LIQ was determined to be liquiritin. Table S1.1: NMR data of compound LIQ and liquiritin. Figure S1.1: UV-VIS spectrum of compound LIQ. Figure S1.2: IR spectrum of compound LIQ. Figure S1.3: APCI-MS spectrum of compound LIQ. Figure S1.4: ^1^H-NMR (500 MHz, CD_3_OD) spectrum of compound LIQ. Figure S1.5: ^13^C-NMR (125 MHz, CD_3_OD) spectrum of compound LIQ. Figure S1.6: DEPT spectrum of compound LIQ. Figure S1.7: HPLC chromatogram of LIQ. Supplemental material 2 (S2): Cellular toxicity. For each group, H9C2 cells were seeded in triplicate into 96-well culture plates at a density of 10^4^ cells per well for 24 h. For testing the cellular toxicity of LIQ, the H9C2 cells was continuously kept in culture medium supplemented LIQ (at dose of 1.2 ÷ 600 *μ*M) for 24 h at 37°C with 5% CO₂. *In vitro* cytotoxicity to LIQ was analyzed by the CCK-8 assay as described in our last study [56]. The absorbance value indicating cell viability was measured at 450 nm using a microplate reader. The number of living cells in each well was expressed as a value relative to the normal control. Experiments were repeated at least 4 times. The cell viability of H9C2 in normal control conditions with and without LIQ supplementation and the IC_50_ value of LIQ were demonstrated in Figure S2.1. Optimum dose of LIQ. We measured protective effect of LIQ on HR-exposed H9C2 cells. For each group, H9C2 cells were seeded in triplicate into 96-well culture plates at a density of 10^4^ cells per well for 24 h. Cells were subjected to hypoxia for 6 h prior to adding different dose of LIQ (1.2 ÷ 300 *μ*M) or NEC (10 *μ*M) to culture media during 24 h reoxygenation. The H9C2 cells of normal control group were cultured in normal incubation chamber for all experimental period. The cell viability under different conditions was analyzed by the CCK-8 assay as described in our last study [56]. Cell viability was assessed by the mitochondrial-dependent reduction of 2-(2-methoxy-4-nitrophenyl)-3-(4-nitrophenyl)-5-(2, 4-disulfophenyl)-2H-tetrazolium, monosodium salt (WST-8) to WST-8 formazan (Cell Counting Kit-8, CCK-8, Dojindo) as described in our last study [56]. The absorbance value indicating cell viability was measured at 450 nm using a microplate reader (Microplate Reader, Molecular Devices, USA). The number of living cells in each well was expressed as a value relative to the normal control. Experiments were repeated at least 3 times. The optimal dose of LIQ in HR-subjected H9C2 model was 60 *μ*M and it was chosen for further experimental assays (Table S2.1, Figure S2.2). Table S2.1: H9C2 cell viability under different LIQ concentrations. Figure S2.1: Cell viability. The graphs indicate H9C2 cell viability after being treated with liquiritin (LIQ) at the doses of 0 ÷ 600 *μ*M (left) and IC_50_ (*μ*M, right) of LIQ in normal control condition. ^*∗*^*p* < 0.05 vs. LIQ-nontreated group; *n* = 4 ÷ 6 for each group. Figure S2.2: The graph indicates H9C2 cell in different conditions. Con: normal control; HR: hypoxia/reoxygenation; LIQ: HR + liquiritin (LIQ); NEC: HR + NecroX-5 (10 *μ*M); ^*∗*^*p* < 0.05 vs. Con; ^#^*p* < 0.05 vs. HR; ^&^*p* < 0.05 vs. LIQ at doses of 1.2, 12, 300, and 600 *μ*M, *n* = 6 for each group.
