# Supplementary material for: Liquiritin from Radix Glycyrrhizae Protects Cardiac Mitochondria from Hypoxia/Reoxygenation Damage
Source: J Anal Methods Chem. 2021 Aug 6;2021:1857464. doi: 10.1155/2021/1857464 (PMC8369190; doi:10.1155/2021/1857464)
Supplement: Supplementary Materials — Supplementation material 1 (S1): Isolation of liquiritin (LIQ) from Radix Glycyrrhizae (liquorice root) (RG). Dried roots of liquorice root (5 kg) were extracted with 96% ethanol (EtOH) under reflux three times (each 20 L). After filtration, the combined EtOH extract was removed under vacuum to obtain a crude extract, which was then suspended in distilled water and partitioned with ethyl acetate (EtOAc) to yield an EtOAc extract (185.0 g). The EtOAc extract was subjected to silica gel column chromatography (CC) and eluted with hexane-EtOAc-methanol (5 : 1 : 0.1, v/v/v), CHCl3-acetone-methanol (3 : 1 : 0.1, v/v/v), and CHCl3-methanol-water (3 : 1 : 0.1, v/v/v) to obtain three main fractions (E1-E3). Fraction E3 (24.5 g) was separated on reversed-phase C18 (RP-C18) CC and eluted with methanol-water (20–70% MeOH, v/v) to afford three subfractions (E3A, E3B, E3C). Subfraction E3B was further purified by RP-C18 CC with methanol-water (40 : 60 v/v) as eluted solvent to yield compound LIQ (441.0 mg). Compound LIQ: yellow powder; mp. 210–212°C; UV (MeOH) λmax 217, 275, 311 nm; IR νmax (cm−1): 3429 (hydroxy), 3329, 2911, 2077, 1607 (carbonyl), 1511, 1446, 1376, 1290, 1233, 1118, 1081, 1044, 997, 889, 834, 632, 531; APCI-MS: m/z 417.2 [M − H]−, 453.1 [M + Cl]− (negative), 419.0 [M + H]+ (positive); C21H22O9; 1H-NMR (500 MHz, CD3OD): δH 7.75 (1H, d, J = 9.0 Hz, H-5), 7,46 (2H, d, J = 9.0 Hz, H-2′, H-6′), 7.16 (2H, d, J = 8.5 Hz, H-3′, H-5′), 6.52 (1H, dd, J = 9.0, 2.5 Hz, H-6), 6.39 (1H, d, J = 2.5 Hz, H-8), 5.47 (1H, dd, J = 13.0, 2.5 Hz, H-2), 4.96 (1H, d, J = 7.0 Hz, H-1″), 3.91 (1H, dd, J = 12.0, 2.0 Hz, H-6″a), 3.71 (1H, dd, J = 12.0, 5.5 Hz, H-6″b), 3.40–3.50 (4H, m, H-2″, H-3″, H-4″, H-5″), 3.06 (1H, dd, J = 17.0, 13.0 Hz, H-3a), 2.75 (1H, dd, J = 17.0, 2.5 Hz, H-3b); 13C-NMR (125 MHz, CD3OD): δC 193.2 (C-4), 166.8 (C-7), 165.4 (C-9), 159.2 (C-4′), 134.4 (C-1′), 129.9 (C-5), 128.8 (C-2′, C-6′), 117.8 (C-3′, C-5′), 115.0 (C-10), 111.8 (C-6), 103.9 (C-8), 102.2 (C-1″), 80 [file 1857464.f1.docx]

**Liquiritin from *Radix Glycyrrhizae* protects cardiac mitochondria from hypoxia/reoxygenation damage**

**Supplementation materials**

**Supplementation material 1 (S1)**

**Isolation of liquiritin (LIQ) from *Radix Glycyrrhizae* (Liquorice root) (RG)**

Dried roots of Liquorice root (5 kg) were extracted with 96% ethanol (EtOH) under reflux three times (each 20 L). After filtration, the combined EtOH extract was removed under vacuum to obtain a crude extract, which was then suspended in distilled water and partitioned with ethyl acetate (EtOAc) to yield an EtOAc extract (185.0 g).

The EtOAc extract was subjected to silica gel column chromatography (CC) and eluted with hexane-EtOAc-methanol (5:1:0.1, v/v/v), CHCl_3_-acetone-methanol (3:1:0.1, v/v/v) and CHCl_3_-methanol-water (3:1:0.1, v/v/v) to obtain three main fractions (E1-E3).

Fraction E3 (24.5 g) was separated on reversed-phase C_18_ (RP-C_18_) CC and eluted with methanol-water (20–70% MeOH, v/v) to afford three subfractions (E3A, E3B, E3C). Subfraction E3B was further purified by RP-C_18_ CC with methanol-water (40:60 v/v) as eluted solvent to yield compound LIQ (441.0 mg).

**Compound LIQ:**

Yellow powder; mp. 210-212°C; UV (MeOH) λ_max_  217, 275, 311 nm; IR ν_max_ (cm^-1^): 3429 (hydroxy), 3329, 2911, 2077, 1607 (carbonyl), 1511, 1446, 1376, 1290, 1233, 1118, 1081, 1044, 997, 889, 834, 632, 531; APCI-MS: *m*/*z* 417.2 [M-H]^-^, 453.1 [M+Cl]^-^ (negative), 419.0 [M+H]^+^ (positive); C_21_H_22_O_9_; ^1^H-NMR (500 MHz, CD_3_OD): *δ*_H_ 7.75 (1H, d, *J* = 9.0 Hz, H-5), 7,46 (2H, d, *J* = 9.0 Hz, H-2′, H-6′), 7.16 (2H, d, *J* = 8.5 Hz, H-3′, H-5′), 6.52 (1H, dd, *J* = 9.0, 2.5 Hz, H-6), 6.39 (1H, d, *J* = 2.5 Hz, H-8), 5.47 (1H, dd, *J* = 13.0, 2.5 Hz, H-2), 4.96 (1H, d, *J* = 7.0 Hz, H-1″), 3.91 (1H, dd, *J* = 12.0, 2.0 Hz, H-6″a), 3.71 (1H, dd, *J* = 12.0, 5.5 Hz, H-6″b), 3.40 - 3.50 (4H, m, H-2″, H-3″, H-4″, H-5″), 3.06 (1H, dd, *J* = 17.0, 13.0 Hz, H-3a), 2.75 (1H, dd, *J* = 17.0, 2.5 Hz, H-3b); ^13^C-NMR (125 MHz, CD_3_OD): *δ*_C_ 193.2 (C-4), 166.8 (C-7), 165.4 (C-9), 159.2 (C-4′), 134.4 (C-1′), 129.9 (C-5), 128.8 (C-2′, C-6′), 117.8 (C-3′, C-5′), 115.0 (C-10), 111.8 (C-6), 103.9 (C-8), 102.2 (C-1″), 80.7 (C-2), 78.2 (C-5″), 78.0 (C-3″), 74.9 (C-2″), 71.4 (C-4″), 62.5 (C-6″), 45.0 (C-3).

The spectral data of LIQ was completely identical with those of liquiritin published in the reference [[1](#_ENREF_1)] (see Table S1.1, Figure S1.1-7); therefore, the compound LIQ was determined to be liquiritin.

**Table S1.1.** NMR data of compound LIQ and Liquiritin

| **No.** | **LIQ** | | **Liquiritin [1]** | |
| --- | --- | --- | --- | --- |
|  | **^1^H-NMR**  **(500 MHz, (CD_3_OD)** | **^13^C-NMR**  **(125 MHz, (CD_3_OD)** | **^1^H-NMR** | **^13^C-NMR**  **(125 MHz, (CD_3_OD)** |
| 2 | 5.47 (1H, dd, 13.0, 2.5) | 80.7 | 5.45 (1H, dd, 12.8, 2.8) | 80.6 |
| 3 | 3.06 (1H, dd, 17.0, 13.0)  2.75 (1H, dd, 17.0, 2.5) | 45.0 | 3.04 (1H, dd, 16.8, 12.8)  2.73 (1H, dd, 16.8, 2.8) | 45.0 |
| 4 | - | 193.2 | - | 193.0 |
| 5 | 7.75 (1H, d, 9.0) | 129.9 | 7.74 (1H, d, 8.8) | 129.7 |
| 6 | 6.52 (1H, dd, 9.0, 2.5) | 111.8 | 6.51 (1H, dd, 8.8, 2.0) | 111.7 |
| 7 | - | 166.8 | - | 166.6 |
| 8 | 6.39 (1H, d, 2.5) | 103.9 | 6.37 (1H, d, 2.0) | 103.7 |
| 9 | - | 165.4 | - | 165.2 |
| 10 | - | 115.0 | - | 114.9 |
| 1′ | - | 134.4 | - | 134.3 |
| 2′, 6′ | 7.46 (2H, d, 9.0) | 128.8 | 7.44 (2H, d, 8.4) | 128.7 |
| 3′, 5′ | 7.16 (2H, d, 8.5) | 117.8 | 7.15 (2H, d, 8.4) | 117.7 |
| 4′ | - | 159.2 | - | 159.0 |
| 1″ | 4.96 (1H, d, 7.0) | 102.2 | 4.95 (1H, d, 7.2) | 102.1 |
| 2″ | 3.40 - 3.50 (4H, m) | 74.9 |  | 74.8 |
| 3″ |  | 78.0 |  | 77.9 |
| 4″ |  | 71.4 |  | 71.3 |
| 5″ |  | 78.2 |  | 78.1 |
| 6″ | 3.91 (1H, dd, 12.0, 2.0)  3.71 (1H, dd, 12.0, 5.5) | 62.5 | 3.91 (1H, dd, 12.0, 1.6)  3.71 (1H, dd, 12.0, 5.6) | 62.5 |

**Figure S1.1.** UV-VIS spectrum of compound LIQ

**
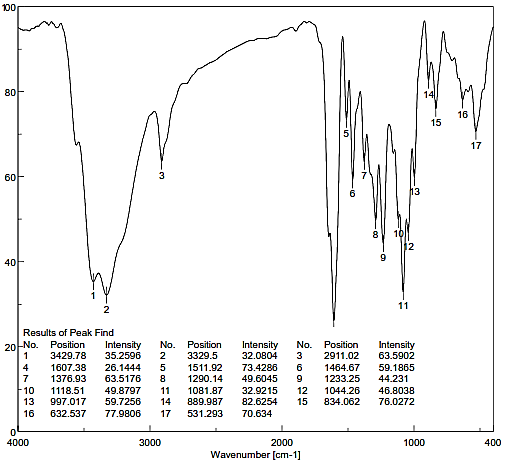
**

**Figure S1.2.** IR spectrum of compound LIQ

**
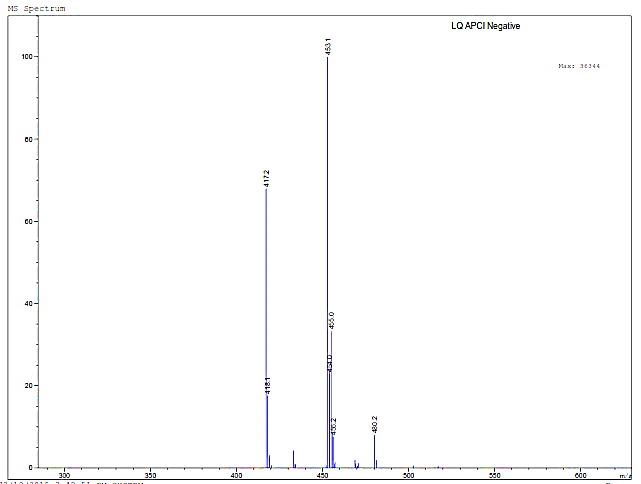

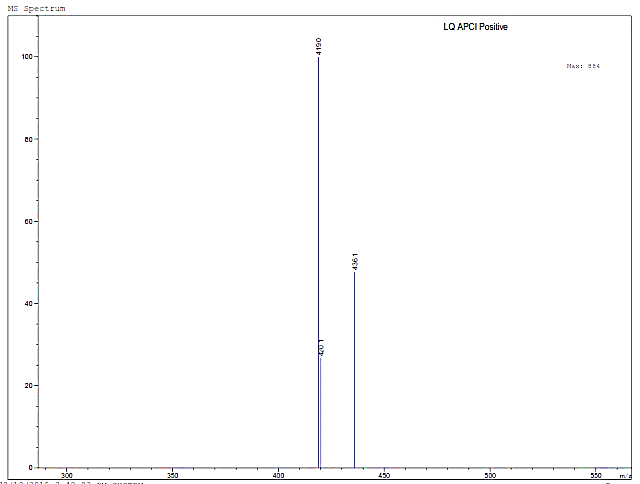
**

**Figure S1.3.** APCI-MS spectrum of compound LIQ

**
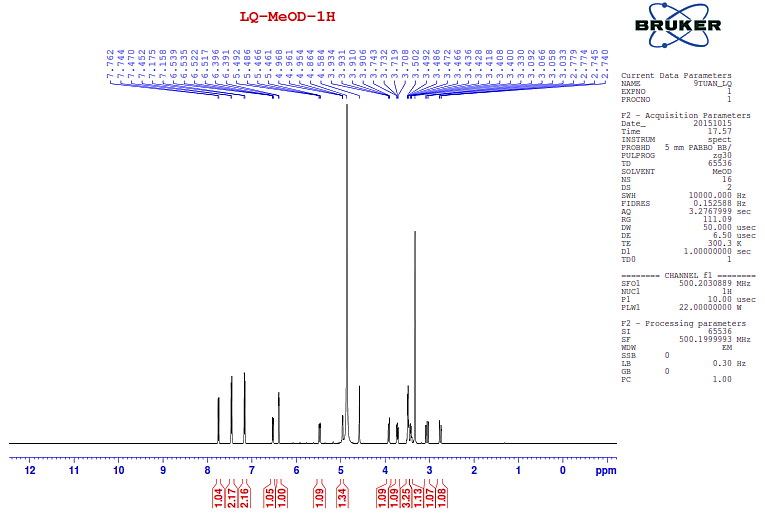
**

**Figure S1.4:** ^1^H-NMR (500 MHz, CD_3_OD) spectrum of compound LIQ

**
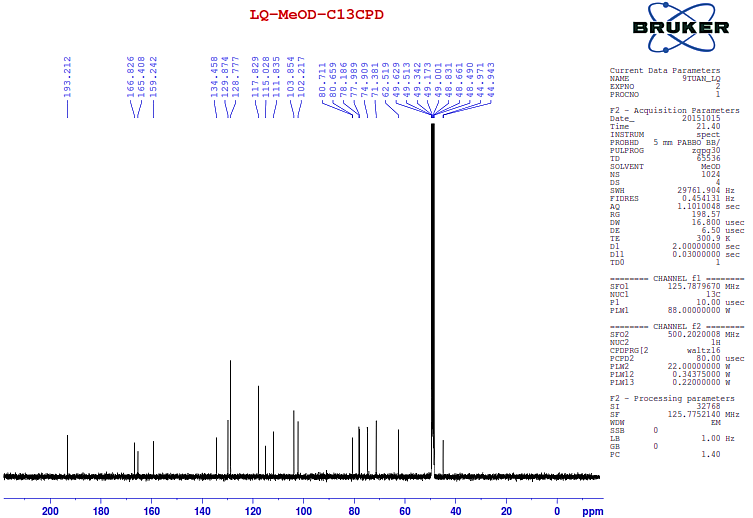
**

**Figure S1.5:** ^13^C-NMR (125 MHz, CD_3_OD) spectrum of compound LIQ

**
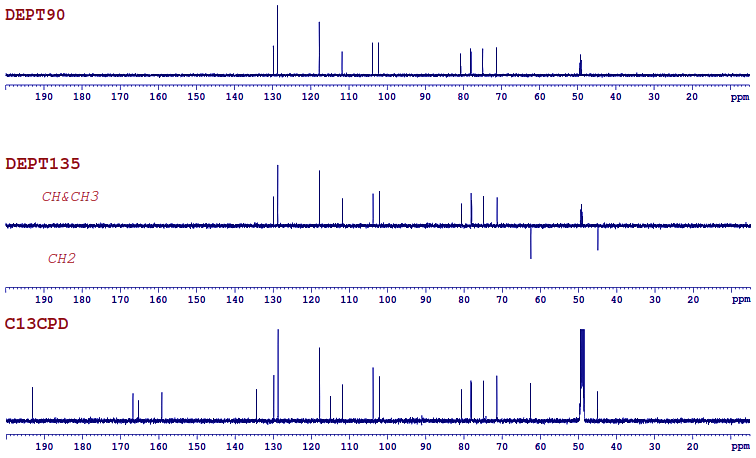
**

**Figure S1.6.** Dept spectrum of compound LIQ

**Figure S1.7.** HPLC Chromatogram of LIQ

**Supplemental material 2 (S2)**

***Cellular toxicity***

For each group, H9C2 cells were seeded in triplicate into 96-well culture plates at a density of 10^4^ cells per well for 24 h. For testing the cellular toxicity of LIQ, the H9C2 cells was continiously kept in culture medium supplemented LIQ (at dose of 1.2÷600 µM) for 24 h at 37°C with 5% CO₂. *In vitro* cytotoxicity to LIQ was analysed by the CCK-8 assay as described in our last study [[2](#_ENREF_2)]. The absorbance value indicating cell viability was measured at 450 nm using a microplate reader. The number of living cells in each well was expressed as a value relative to the normal control. Experiments were repeated at least 4 times. The cell viablity of H9C2 in normal control conditions with and without LIQ supplementation and the IC_50_ value of LIQ were demonstrated in Figure S2.1.

***Optimum dose of LIQ***

We measured protective effect of LIQ on HR-exposed H9C2 cells. For each group, H9C2 cells were seeded in triplicate into 96-well culture plates at a density of 10^4^ cells per well for 24 h. Cells were subjected to hypoxia for 6 h prior to adding different dose of LIQ (1.2÷300 µM) or NEC (10 µM) to culture media during 24 h reoxygenation. The H9C2 cells of normal control group were cultured in normal incubation chamber for all experimental period. The cell viability under different conditions was analysed by the CCK-8 assay as described in our last study [[2](#_ENREF_2)]. Cell viability was assessed by the mitochondrial-dependent reduction of 2-(2-methoxy-4-nitrophenyl)-3-(4-nitrophenyl)-5-(2, 4-disulfophenyl)-2H-tetrazolim, monosodium salt (WST-8) to WST-8 formazan (CCK-8, Dojindo) as described in our last study [[2](#_ENREF_2)]. The absorbance value indicating cell viability was measured at 450 nm using a microplate reader (Microplate Reader, Molecular Devices, USA). The number of living cells in each well was expressed as a value relative to the normal control. Experiments were repeated at least 3 times. The optimal dose of LIQ in HR-subjected H9C2 model was 60 µM and it was chosen for further experimental assays (Table S2.1, Figure S2.2).

***Table S2.1.*** *The H9C2 cell viablity under different LIQ concentrations*

| **Cell viability**  **LIQ (µM)** | **MEAN±SEM**  **(%)** |
| --- | --- |
| **1.2** | **100.02±0.45** |
| **1.2** | **103.38±1.25** |
| **12** | **101.18±0.93** |
| **60** | **108.31±2.80** |
| **120** | **104.39±2.48** |
| **300** | **94.52±1.92** |
| **600** | **26.59±1.25** |

**
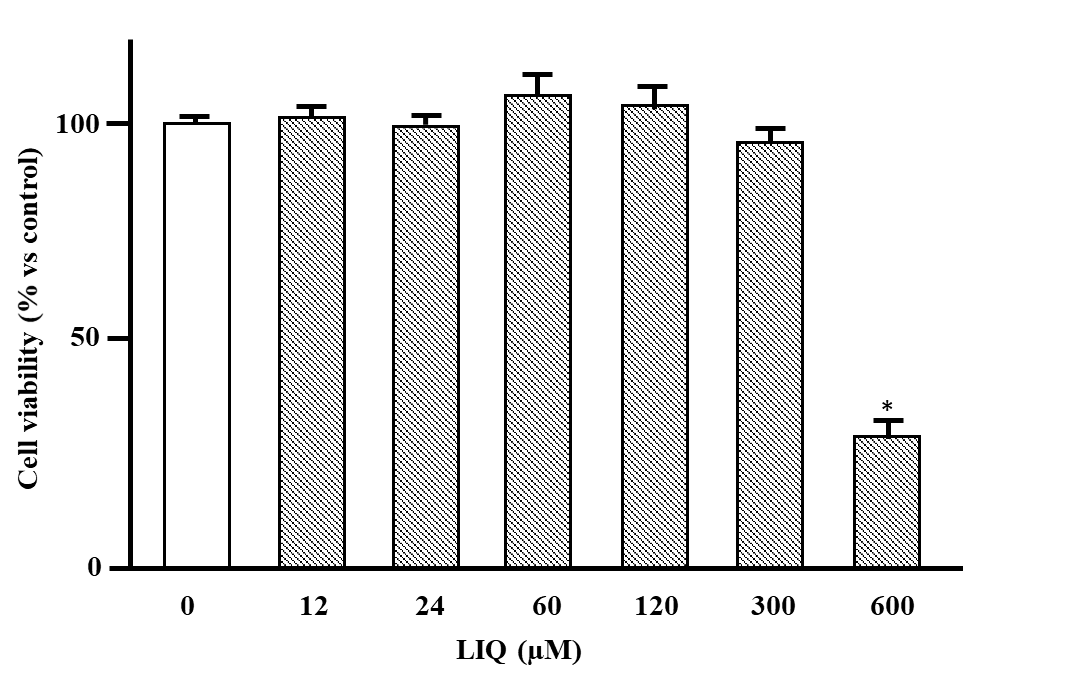

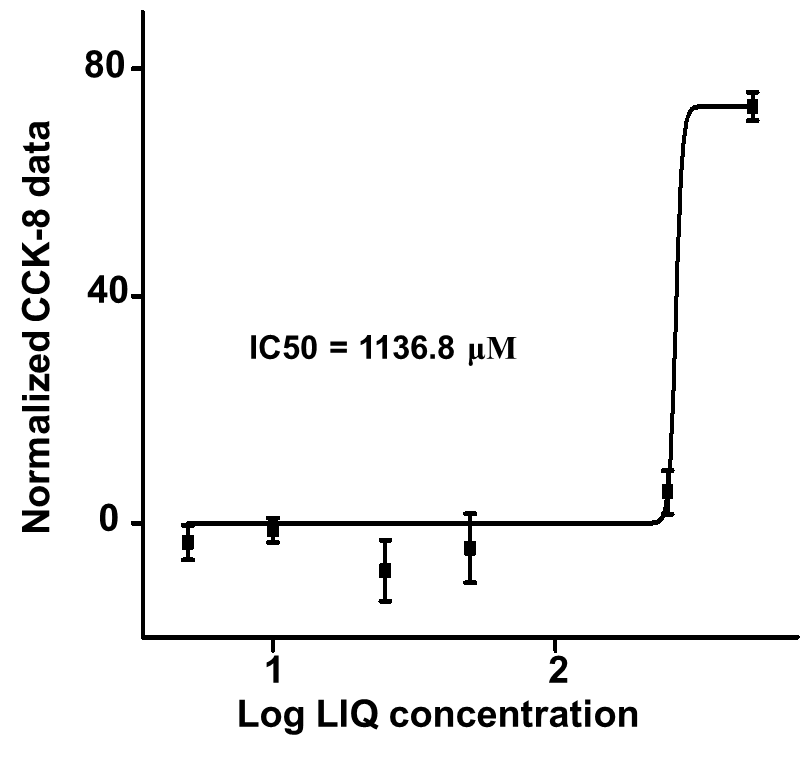
**

**Figure S2.1.** Cell viability. The graphs indicate H9C2 cell viability after treated with Liquiritin (LIQ) at the doses of 0÷600 µM (left) and IC_50_ (µM, right) of LIQ in normal control condition. **p* < 0.05 vs. LIQ-nontreated group; n = 4÷6 for each group.

**
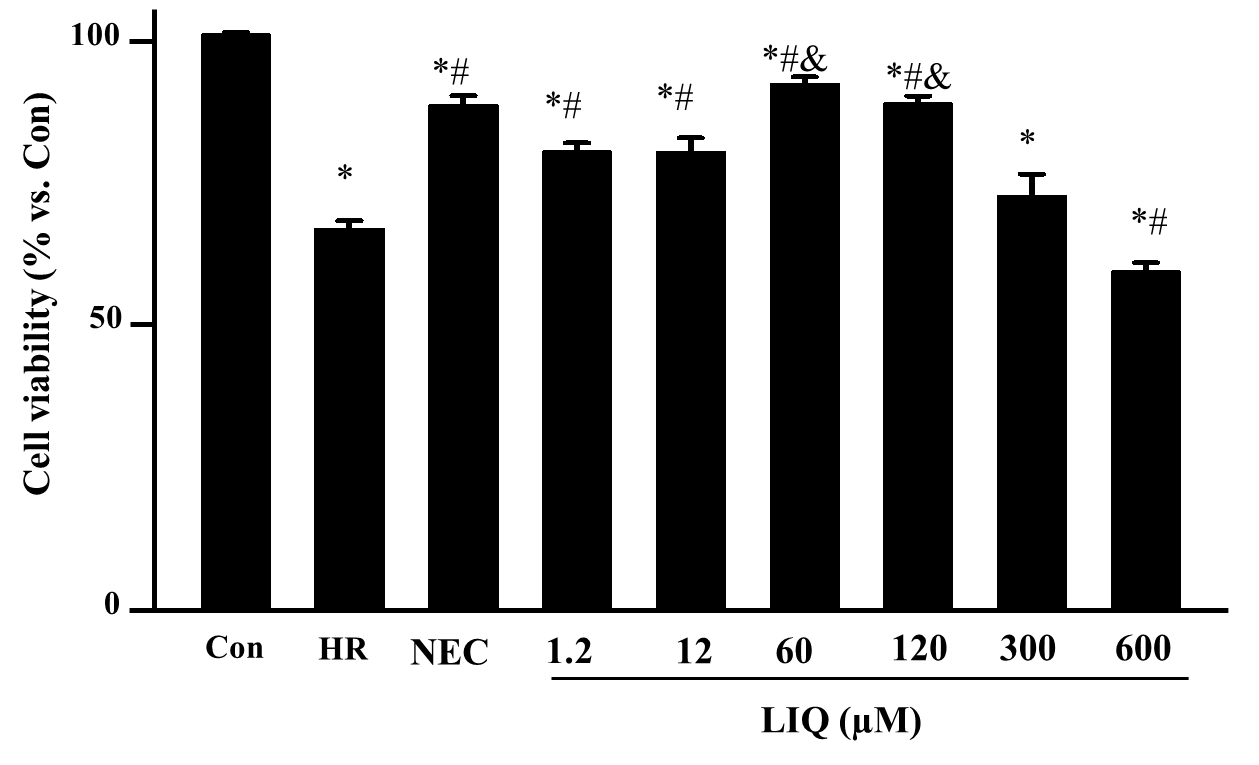
**

**Figure S2.2.** The graph indicates H9C2 cell in different conditions. Con: normal control; HR: hypoxia/reoxygenation; LIQ: HR + Liquiritin (LIQ); NEC: HR + NecroX-5 (10 µM); **p* < 0.05 vs. Con; ^#^*p* < 0.05 vs. HR; ^&^*p* < 0.05 vs. LIQ at doses of 1.2, 12, 300 and 600 µM, n = 6 for each group.

**References**

1. Abd El Azim, M., et al., *Anti-tumor, antioxidant and antimicrobial and the phenolic constituents of Clove Flower Buds (Syzygium aromaticum).* Journal of Microbial & Biochemical Technology, 2014.

2. Thu, V.T., et al., *NecroX-5 exerts anti-inflammatory and anti-fibrotic effects via modulation of the TNFα/Dcn/TGFβ1/Smad2 pathway in hypoxia/reoxygenation-treated rat hearts.* The Korean Journal of Physiology & Pharmacology : Official Journal of the Korean Physiological Society and the Korean Society of Pharmacology, 2016. **20**(3): p. 305-314.
